# Does continuity in nursing staff matter? A pilot study on correlation of central line-associated bloodstream infections and employee turnover

**DOI:** 10.1186/s13756-021-00958-z

**Published:** 2021-06-06

**Authors:** Thomas Scheier, Stefan P. Kuster, Mesida Dunic, Christian Falk, Hugo Sax, Peter W. Schreiber

**Affiliations:** 1 Division of Infectious Diseases and Hospital Epidemiology, University Hospital Zurich and University of Zurich, Raemistrasse 100, 8091 Zurich, Switzerland; 2 Information and Communication Technology, University Hospital Zurich, Zurich, Switzerland

**Keywords:** CLABSI, Personnel turnover, Surveillance, Hospital-acquired infections, Infection prevention, Infection control

## Abstract

**Background:**

Understaffing has been previously reported as a risk factor for central line-associated bloodstream infections (CLABSI). No previous study addressed the question whether fluctuations in staffing have an impact on CLABSI incidence. We analyzed prospectively collected CLABSI surveillance data and data on employee turnover of health care workers (HCW) to address this research question.

**Methods:**

In January 2016, a semiautomatic surveillance system for CLABSI was implemented at the University Hospital Zurich, a 940 bed tertiary care hospital in Switzerland. Monthly incidence rates (CLABSI/1000 catheter days) were calculated and correlations with human resources management-derived data on employee turnover of HCWs (defined as number of leaving HCWs per month divided by the number of employed HCWs) investigated.

**Results:**

Over a period of 24 months, we detected on the hospital level a positive correlation of CLABSI incidence rates and turnover of nursing personnel (Spearman rank correlation, *r* = 0.467, *P* = 0.022). In more detailed analyses on the professional training of nursing personnel, a correlation of CLABSI incidence rates and licensed practical nurses (Spearman rank correlation, *r* = 0.26, *P* = 0.038) or registered nurses (*r* = 0.471, *P* = 0.021) was found. Physician turnover did not correlate with CLABSI incidence (Spearman rank correlation, *r* =  −0.058, *P* = 0.787).

**Conclusions:**

Prospectively determined CLABSI incidence correlated positively with the degree of turnover of nurses overall and nurses with advanced training, but not with the turnover of physicians. Efforts to maintain continuity in nursing staff might be helpful for sustained reduction in CLABSI rates.

## Introduction

A recent US point prevalence study found that approximately 3% of all hospitalized patients had a healthcare-associated infection (HAI) [1]. The most frequent HAIs were pneumonia, gastrointestinal infections and surgical site infections. Central line-associated bloodstream infections (CLABSI) were reported in 0.30% of all hospitalized patients.

CLABSI contribute to major morbidity, e.g. septic complications such as osteomyelitis, distant abscess formation or endocarditis, and are characterized by a relevant case fatality rate. Stevens et al. reported a 2.27-fold increased risk of mortality for patients suffering from CLABSI [2]. Besides negative impact on patient outcomes, catheter-related bloodstream infections are costly hospital-acquired infections with an attributable cost between 4′200 and 13′030 € in Europe [3].

For CLABSI prevention, bundle approaches have proven to be efficient and are broadly used [4]. Common bundle components include full barrier precautions at the time of insertion, use of chlorhexidine for skin antisepsis, avoidance of the femoral site, removal of unnecessary catheters and checklists. Fridkin et al.identified patient-to-nurse ratio as an independent risk factor for CLABSI [5]. Current guidelines also highlight the relevance of staffing [6, 7], e.g. Marschall et al. indicate an adequate patient-to-nurse ratio as a component in CLABSI prevention [7]. However, the role of employee turnover on CLABSI incidence has been not investigated up to now. We aimed at this research question using prospectively collected data on CLABSI incidence and human resources management derived data on personnel turnover of health care workers.

## Material and methods

### Setting

We performed this study at the University Hospital Zurich, a 940 bed tertiary care center with approximately 40,000 admission per year featuring all medical specialties except pediatrics and orthopedics. At the study hospital, the established CLABSI prevention bundle encompassed aspects of the catheter insertion site, the process of catheter insertion, skin disinfection, dressing change, infusion handling, and a list of accepted CVC indications. Every six months, CLABSI rates were reported for the whole hospital and for each institute or department. On intensive care and intermediate care units, daily chlorhexidine bathing was implemented for patients with a central line in the third quarter of 2017. Chlorhexidine bathing was performed by registered nurses (RN) or licensed practical nurses (LPN). Central lines are exclusively inserted by physicians with assistance of RN. Catheter maintenance, dressing changes, drawing of blood cultures, administration of drugs and catheter removal is limited to RN on normal wards and intermediate care units, whereas LPN are also allowed to perform dressing changes and catheter removal on intensive care units.

### Surveillance of central line-associated bloodstream infections

Since January 2016 the team of infection prevention and control has been equipped with a semiautomatic surveillance system for CLABSI incidence. For this application, the variables presence of a central venous catheter, length of hospitalization, and microbial results of blood cultures are continuously extracted from the patient data management system into Caradigm Intelligence Platform® (CIP). CIP is used as platform for data aggregation and interpretation. Within CIP the data is subjected to an algorithm based on Centers for Disease Control and Prevention (CDC) CLABSI definitions. As a remaining manual step in CLABSI surveillance, a list of individuals with possible CLABSI needs to be verified. An infection control nurse manually excludes patients with positive blood cultures resulting from contamination or secondary bacteremia originating from another site of infection. Throughout the study period, a single infection control nurse was responsible to verify CLABSI. For the current study, we used CLABSI data encompassing two years (2016 and 2017), which were used to calculate monthly incidence rates reported as CLABSI/1000 catheter days.

### Human resources management derived data

The human resources management of the University Hospital Zurich provided data on the number of leaving nurses and physicians per month and the corresponding number of health care workers (HCW) employed per month. For nurses detailed information on the level of professional training was available, i.e. assistive nursing personnel (ANP), “Fachangestellte Gesundheit”, which was considered as a correlate of licensed practical nurse (LPN), or registered nurse (RN). Information on the employment level of physicians, i.e. assistant physician, attending physician or senior physician, was provided. Employee turnover was calculated by dividing the number of leaving HCW by the number of currently employed HCWs of the corresponding profession.

### Statistical analysis

All statistical analyses were performed in R (version 3.5.0; R Foundation for Statistical Computing, Vienna, Austria). Incidence rates were determined by dividing the number of CLABSI by number of catheter days in the corresponding time period and reported as CLABSI/1000 catheter days. In general, two-sided tests were used; *p*-values ≤ 0.05 were considered statistically significant. For investigation of linear relationships between two variables, we applied Spearman rank correlation. For correlation analyses monthly data for CLABSI incidence rates and personal turnover of HCW were used. To address the relevance of the professional training, subset analyses for ANP, LPN, and RN as well as for the employment levels of physicians were added.

## Results

### Incidence of central line-associated bloodstream infections

**Fig. 1:**
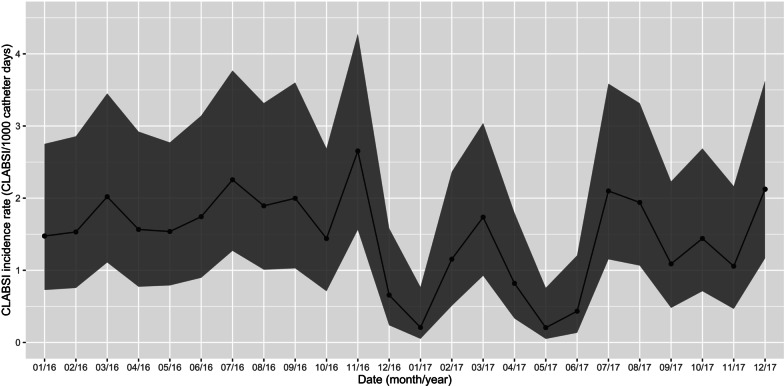
Incidence rates of central line-associated bloodstream infections on hospital level. The x-axis indicates the date (month/year). The y-axis displays the incidence rate of CLABSI. Dots correspond to monthly means and dark gray area to 95% confidence intervals

Over a period of 24 months a total of 167 central line-associated infections (CLABSI) was observed. Median monthly CLABSI incidence was 1.53 CLABSI/1000 catheter days (interquartile range (IQR) 1.08–1.96 CLABSI/1000 catheter days) (Fig. 1).

### Personnel turnover

**Fig. 2:**
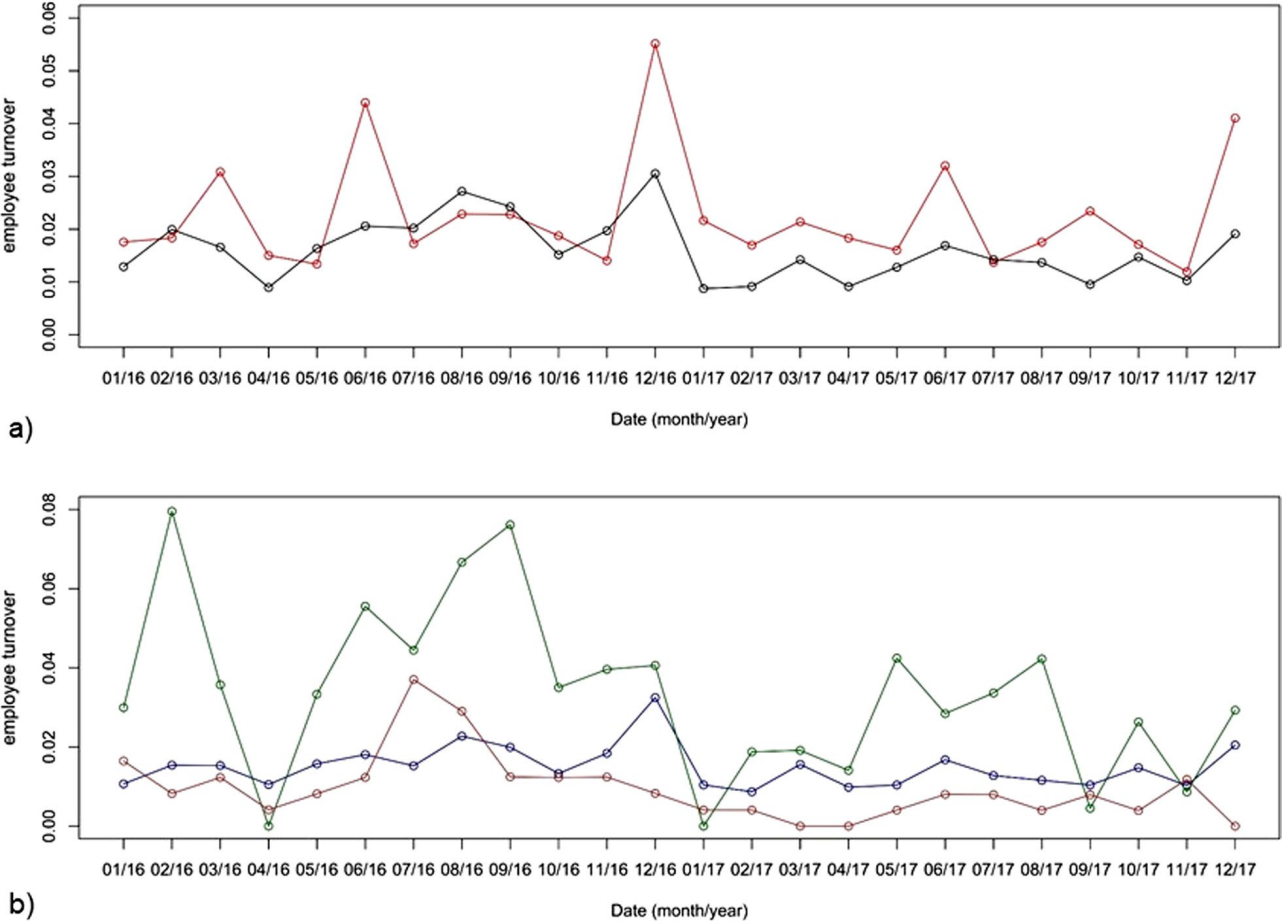
Employee turnover during study period on hospital level. **a** Employee turnover of physicians (red line) and overall nursing personnel (black line); **b** employee turnover of assistive nursing personnel (brown line), licensed practical nurses (green line), and registered nurses (blue line); The x-axis indicates the date (month/year). The y-axis displays the employee turnover (number of leaving HCWs divided by the number of currently employed HCWs of the corresponding profession)

Monthly median turnover of overall nursing personnel was 0.015 (IQR 0.012–0.020) (Fig. 2a), 0.008 (IQR 0.004–0.012) for ANP, 0.034 (IQR 0.019–0.042) for LPN and 0.015 (IQR 0.011–0.017) for RN (Fig. 2b), respectively. For all physicians median turnover was 0.018 (IQR 0.017–0.023) per month (Fig. 2a). Considering the employment level of physicians, median monthly turnover was 0.026 (IQR 0.023–0.032) for assistant physicians, 0.011 (IQR 0.007–0.017) for attending physicians and 0.008 (IQR 0.000–0.011) for senior physicians.

### Correlation of central line-associated bloodstream infections and personnel turnover

**Fig. 3:**
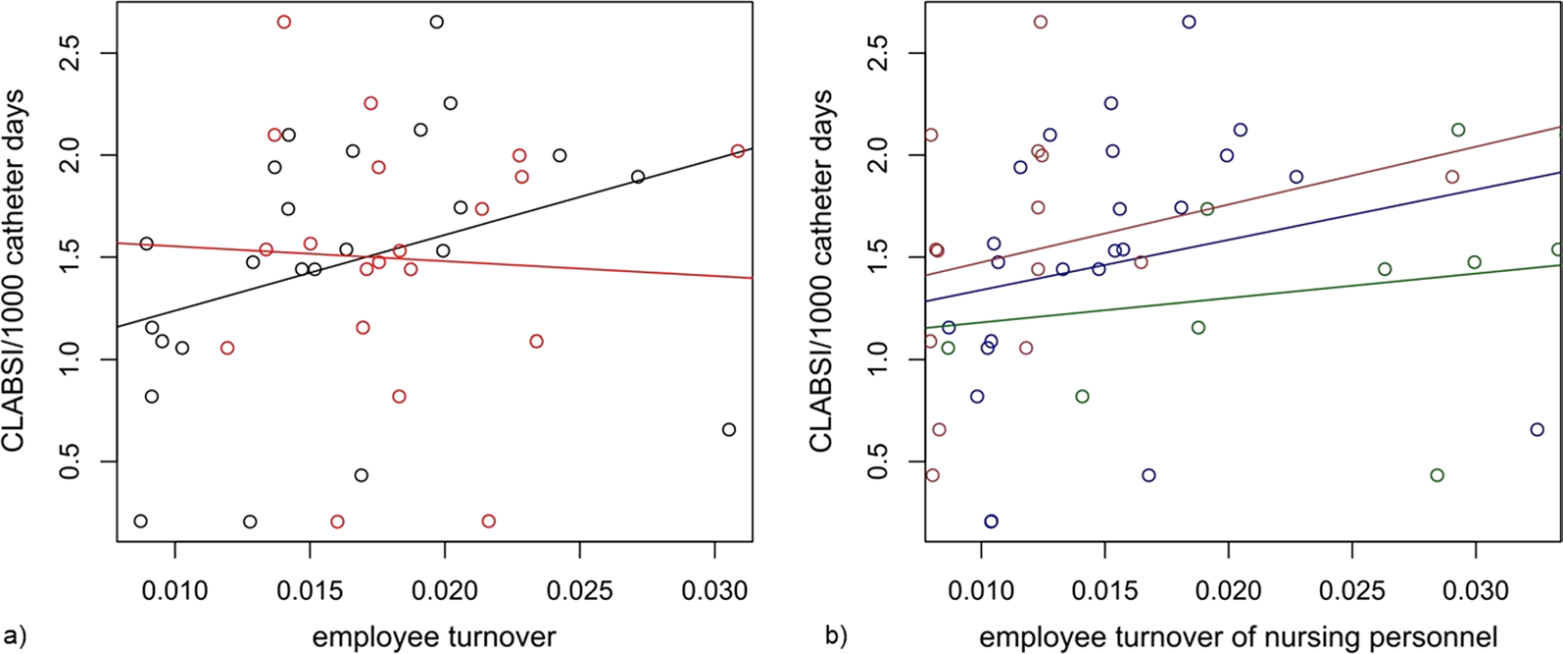
Correlation of personnel turnover and CLABSI incidence on hospital level. **a** Correlation of CLABSI incidence and turnover of overall nursing personnel (black line; *r* = 0.467, *P* = 0.022) and physicians (red line; *r* =  −0.05, *P* = 0.787), respectively; **b** correlation of CLABSI incidence and turnover of assistive nursing personnel (brown line; *r* = 0.32, *P* = 0.122), licensed practical nurses (green line; *r* = 0.26, *P* = 0.038), and registered nurses (blue line; *r* = 0.471, *P* = 0.021), respectively; The x-axis indicates employee turnover (number of leaving HCWs divided by the number of currently employed HCWs of the corresponding profession). The y-axis displays the incidence rate of CLABSI. Lines were generated using linear regression

On the hospital level, a positive correlation of CLABSI incidence and nursing personnel turnover was detected (*r* = 0.467, *P* = 0.022) (Fig. 3a). The positive correlation with CLABSI was confirmed for the subgroup of RN (*r* = 0.471, *P* = 0.021) and LPN (*r* = 0.426, *P* = 0.038), but not for ANP (*r* = 0.324, *P* = 0.122) (Fig. 3b).

For physician turnover we did not observe a significant correlation with CLABSI incidence (*r* =  −0.05, *P* = 0.787) (Fig. 3a) independent of the employment level (assistant physicians *r* =  −0.17, *P* = 0.436; attending physicians *r* = 0.30, *P* = 0.148; senior physicians *r* = 0.03, *P* = 0.874).

## Discussion

In our prospective study encompassing 24 months, we found a positive correlation of CLABSI incidence and turnover of nursing personnel on the hospital level. Among nursing personnel, the positive correlation was confirmed for LPNs and RNs, but not for ANPs. No significant correlation was detected for physician turnover.

CLABSI can result from contamination of infusion systems or more frequently from catheter contamination [8]; especially, the catheter hub seems to be a vulnerable location in contamination of central lines [9]. Causative pathogens originate either from the patient’s flora itself, the hands of HCWs or contaminated medical products, e. g. disinfectants used for skin cleansing. We found a positive correlation of CLABSI incidence and turnover of nurses with advanced training, i.e. LPN and RN, which reflects the subset of nursing personnel that is involved in central line handling. Robert et al. highlighted the role of continuity in nursing staff in primary bacteremias [10]. Composition of nurse staff defined as pool-nurse-to-patient-ratio was found to be an independent risk factor for CLABSI with an odds ratio of 3.8. Similarly, our study supports a relevance of continuity in nursing personnel for CLABSI prevention.

For physicians we did not detect an association of turnover and CLABSI incidence. The finding that an association was exclusively detected with nursing staff turnover, but not with physician turnover might reflect the crucial role of catheter care and infusion management after central line placement. At our center, central lines are exclusively placed by physicians, whereas later on central line care and intravenous drug administration is provided by nurses. It can be speculated that prolonged work experience at a specific hospital or even ward results in improved knowledge of infection prevention measures and center specific central line products thus lowering the risk of CLABSI. Overall nursing experience might be also relevant for prevention of HAIs in general. One hypothesis can be that a parallel increase in infection prevention skills goes along with duration of employment as nurse. Nursing staff inexperience has been associated with a negative impact on quality of care in ICUs indicated by the frequency and outcome of incidents related to inexperience [11]. However, in our dataset we did not have information on the individual’s work experience hindering an analysis on the influence of fluctuations in overall nursing experience. Studies on the role of nursing personnel turnover in HAIs are scarce. In a multicenter study encompassing 59 US nursing homes, turnover of RNs was associated with a higher incidence of infectious complications as well as infection-related hospitalization [12]. The authors speculated that personnel turnover makes an establishment and maintenance of effective infection control practices difficult. Turnover could result in inconsistencies in supervision and training which might affect quality.

The level of professional training among nursing personnel has been previously reported as relevant for patient outcomes. Needleman et al. found that a larger proportion of care provided by RNs was associated with a reduction of urinary tract infections and pneumonia among medical patients. A larger proportion of care provided by RNs correlated with a decrease in urinary tract infections among surgical patients [13]. Similarly, in an US study an increase in the ratio of licensed nurses to total nursing staff correlated with a lower incidence of pneumonia [14]. Among trauma patients a higher ratio of nursing hours provided by LPNs to total nursing hours provided by LPNs and RNs showed a significantly higher odds in mortality and HAIs [15]. Recent data also support a protective effect from academic training among nurses. In a European study, an increase in nurses with a bachelor degree was associated with lower in-hospital mortality [16]. The steady rising cost pressure likely impacts composition of nurse staffing. To save costs nursing activities might be shifted from higher qualified nursing personnel such as RNs or LPNs to ANPs. However, as outlined above these changes could affect the likelihood of adverse patient outcomes such as HAIs. Future studies that address economic aspects of care should consider both, the higher salary for better qualified nursing personnel and the additional costs for CLABSI.

Strengths of the present study include the prospective design with standardized CLABSI surveillance ensuring strict application of CDC definitions and the availability of monthly data on personnel turnover.

Our study has several limitations. We were not able to address numerous potentially confounding variables for several reasons. The study duration was limited to 2 years as changes in the patient data management system of the intensive care units put our semiautomatic CLABSI surveillance system out of order. This restricted study duration hampered statistical analyses, as due to the limited study duration the number of CLABSI was small for several institutes or departments. In addition, the granularity of certain variables was limited, as e.g. HRM-derived data was aggregated at the level of institute or department. These limitations did not allow to perform detailed analyses per ward. Furthermore, we did not have information on nurse:patient ratios and on the use of pool or float nurses.

However, our pilot study should motivate to plan larger-scale studies with the possibility to correct for these confounders in future and allow more detailed analysis. Future studies, ideally with multi-centric design, that investigate the role of nursing personnel turnover on CLABSI incidence and other HAIs seem warranted.

## Data Availability

Please contact author for data request.

## Abbreviations

ANP: Assistive nursing personnel
CDC: Centers for Disease Control and Prevention
CLABSI: Central line-associated bloodstream infection
HAI: Healthcare associated infection
HCW: Health care worker
IQR: Inter quartile range
LPN: Licensed practical nurse
RN: Registered nurse
